# Synthesis of mesogenic phthalocyanine-C_60_ donor–acceptor dyads designed for molecular heterojunction photovoltaic devices

**DOI:** 10.3762/bjoc.5.49

**Published:** 2009-10-07

**Authors:** Yves Henri Geerts, Olivier Debever, Claire Amato, Sergey Sergeyev

**Affiliations:** 1 Université Libre de Bruxelles (ULB), Laboratoire de Chimie des Polymères, CP 206/01, Boulevard du Triomphe, 1050 Bruxelles, Belgium; 2 University of Antwerp, Department of Chemistry, Groenenborgerlaan 171, 2020 Antwerp, Belgium

**Keywords:** donor–acceptor dyad, fullerene, liquid crystals, phthalocyanine, phthalonitrile

## Abstract

A series of phthalocyanine-C_60_ dyads **2a–d** was synthesized. Key steps in their synthesis are preparation of the low symmetry phthalocyanine intermediate by the statistical condensation of two phthalonitriles, and the final esterification of the fullerene derivative bearing a free COOH group. Structural characterization of the molecules in solution was performed by NMR spectroscopy, UV–vis spectroscopy and cyclic voltammetry. Preliminary studies suggest formation of liquid crystalline (LC) mesophases for some of the prepared dyads. To the best of our knowledge, this is the first example of LC phthalocyanine-C_60_ dyads.

## Introduction

Among sustainable energy technologies, photovoltaic (PV) conversion of solar energy is considered as a promising solution. Although currently the market is dominated by inorganic PV devices, development of organic PV materials is driven by their compatibility with solution processing and hence the potentially low cost of large-scale production by printing technologies [1–3]. One of the most popular concepts in the design of organic PV devices is the “bulk heterojunction” architecture, featuring blends of the two immiscible materials: a donor and an acceptor of electrons [4–7]. After absorption of a photon, an initially formed exciton is dissociated at the donor/acceptor interface into a positive charge (hole) and a negative charge (electron), which are transported to the electrodes. Hence, a critical issue in bulk heterojunction PV devices is the control of morphology of materials, in order to provide both the efficient exciton generation and the rapid charge carrier transport. The logical step in the development of this architecture is “molecular heterojunction”, that is, creation of covalent linkages between donor and acceptor components [6]. The main idea is that in material well-organized on the molecular level, donor and acceptor moieties will create the separate pathways for the transport of holes and electrons, respectively. One can intuitively expect that for such high order of self-organization, liquid crystalline (LC) materials will be beneficial, because they combine order and fluidity, which allows the self-healing of structural defects [8].

**Figure 1:**
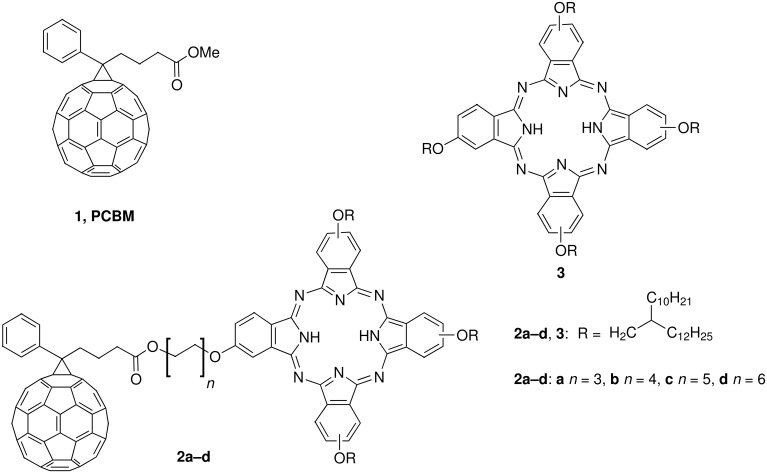
Phthalocyanine-C_60_ dyads **2a–d** described in this paper, C_60_-derivative **1** (PCBM) and previously reported mesogenic phthalocyanine **3**.

One of the most widely used molecules in the bulk heterojunction PV devices is fullerene C_60_, firstly in the pristine form and later as easily soluble derivatives such as phenylcyclopropa[6,6]C_60_-butanoic acid methyl ester (PCBM, **1**, Figure 1) [9–12]. It has been used in a combination with a plethora of conjugated polymers [13–18] as well as small molecules [19–21]. Furthermore, a number of C_60_-containing covalent ensembles were studied within the “molecular heterojunction” concept [22–29].

Phthalocyanines (Pc) have found a number of industrial applications as dyes and pigments due to their bright blue or green colors combined with extraordinary thermal and photochemical stability [30]. Phthalocyanines bearing flexible peripheral substituents form columnar mesophases [8,31], which demonstrate very efficient charge transport along the columns [32–33]. A unique combination of properties makes phthalocyanines excellent candidates as active materials for photovoltaic devices.

Within the concept of a “molecular heterojunction”, a number of phthalocyanine-fullerene dyads and triads were synthesized [34–38] and long-lived photoinduced charge transfer was demonstrated in these type of materials [39]. Although examples of mesogenic fullerene derivatives have been previously reported [40–44], mesogenic Pc-C_60_ dyads are unknown, most probably due to the tedious purification of the unsymmetrically substituted phthalocyanines bearing long peripheral substituents. A possible elegant solution to this problem was demonstrated by Torres and co-workers, who obtained LC mesophases of several phthalocyanine-C_60_ dyads by blending them with a mesogenic phthalocyanine derivative [45]. In this paper we report the synthesis of the first example, to the best of our knowledge, of mesogenic phthalocyanine-fullerene dyads.

## Results and Discussion

The structure of dyads **2a–d** is depicted in Figure 1. We decided to combine in one molecule the phenylcyclopropa[6,6]C_60_ moiety of PCBM as an acceptor and a phthalocyanine bearing three swallow-tail alkoxy groups as a donor. The choice of the substituents on the phthalocyanine core was dictated by the previous finding that the pseudosymmetrical phthalocyanine **3** bearing four such substituents is very soluble in many organic solvents, forms columnar mesophases over a broad temperature range and, at the same time, possesses a reasonably low clearing point (ca. 180 °C) [46]. Finally, a critical issue in obtaining a mesogenic phthalocyanine-C_60_ dyad can be a difficulty in accommodating a bulky C_60_ molecule in the columnar LC mesophases [45]. To this end, we envisaged the variation of the linker between the fullerene and the phthalocyanine unit.

**Scheme 1:**
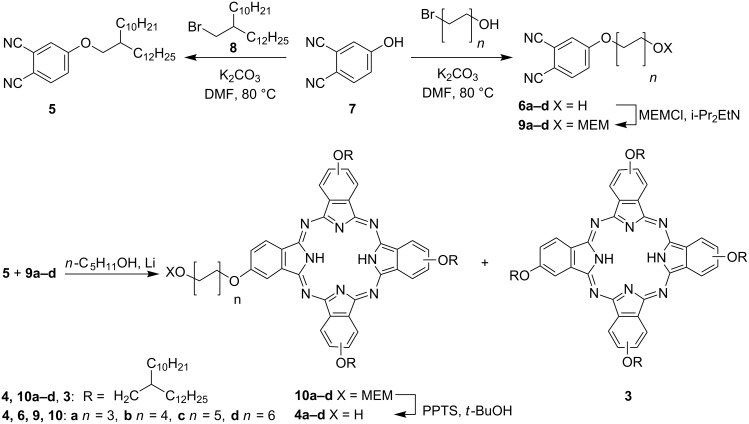
Synthesis of low-symmetry phthalocyanines **4a–d**.

**Scheme 2:**
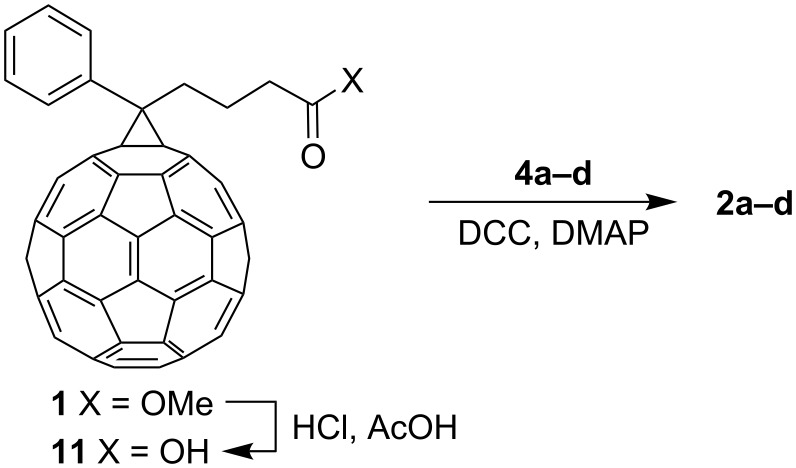
Synthesis of dyads **2a–d**.

The synthesis of dyads **2a–d** is summarized in Scheme 1 and Scheme 2. The key step, which also represents a bottle-neck in terms of yield (see below), is the preparation of the low-symmetry phthalocyanines **4a–d**. To this end, we originally decided to explore the statistical condensation of phthalonitriles **5** and **6a–d** (Scheme 1). Previously, synthesis of 4-alkoxyphthalonitriles such as **5** via aromatic nucleophilic substitution in 4-nitrophthalonitrile with the corresponding alcohols in the presence of LiOH in DMSO was reported [46]. However, the yields of phthalonitriles **5** were moderate (below 50%) and their separation from multiple side products was cumbersome. Hence, we preferred to prepare **5** (80% yield) by Williamson reaction between phenol **7** and bromide **8**. The latter was synthesized from the corresponding commercially available alcohol by the well-known general method (CBr_4_/PPh_3_) [47]. A similar procedure was then used to synthesize phthalonitriles **6a–d** (80–86% yield) starting from the corresponding commercially available ω-bromoalcohols.

Several strategies for the synthesis of low-symmetry A_3_B phthalocyanines (where A and B refer to the chemically different isoindole units comprising the phthalocyanine core) were reported earlier, including expansion of subphthalocyanines or solid-phase synthesis on polymer supports [48]. However, operationally simplest and shortest route is the statistical condensation of the two different phthalonitriles. This method inevitably produces a mixture of the desired A_3_B phthalocyanines with the products of compositions A_4_, A_2_B_2_, AB_3_ and B_4_. In theory, 3 : 1 molar ratio of the two dinitriles is required to access A_3_B products. However, using larger excess of one of dinitriles (often 9 : 1 stoichiometry is used) is more practical. Although the amount of the A_4_ phthalocyanine in the reaction mixture grows, the formation of cross-condensation products other than A_3_B is essentially suppressed (see Figure SI2 in the Supporting Information for the graphical presentation of the product distribution). Hence, the isolation of the desired A_3_B is limited to the separation from the A_4_. Another factor that facilitates the separation by chromatography is rather different polarity of the substituents on A and B units. In our case, the oligomethylene linker with a terminal OH group is much more polar than the bulky swallow-tail alkyl group, and we did not expect difficulties in the separation of condensation products.

However, attempted condensation of **5** and **6a–d** gave no desired phthalocyanine derivative. We reasoned that the unprotected OH group in one of the reaction components may affect the condensation, and converted alcohols **6a–d** into acetals **9a–d** (84–93% yield) by treatment with an excess of methoxyethoxymethyl chloride (MEMCl) in the presence of *i*-Pr_2_EtN as a base. The general choice of acetal as a protective group was dictated by its excellent stability in the presence of the strong bases, required for the cyclotetramerization of phthalonitriles. Among various popular acetal protective groups, the relatively polar MEM moiety was chosen to facilitate the chromatographic separation of the unsymmetrical phthalocyanines **10a–d** from the major side product **3**: the latter bears only relatively apolar, bulky swallow-tail alkoxy groups. In addition, the signal of the terminal methyl group of the MEM moiety serves as a convenient “marker” in rather complex ^1^H NMR spectra of phthalocyanines **10a–d** (see below). Condensation of **5** and **9a–d** in *n*-C_5_H_11_OH/*n*-C_5_H_11_OLi upon reflux afforded the desired A_3_B phthalocyanines **10a–d** (13–24% after column chromatography) together with pseudo-symmetrical A_4_ phthalocyanine **3** (35–51%). The seemingly modest yields of **10a–d** actually correspond to those reported earlier for other A_3_B-type phthalocyanines: yields higher than 20% are rare in such reactions [45,49–51]. The MEM protective group in **10a–d** was then quantitatively removed to give **4a–d** upon treatment with pyridinium tosylate (PPTS) in *t*-BuOH according to previously reported general method [52].

**Figure 2:**
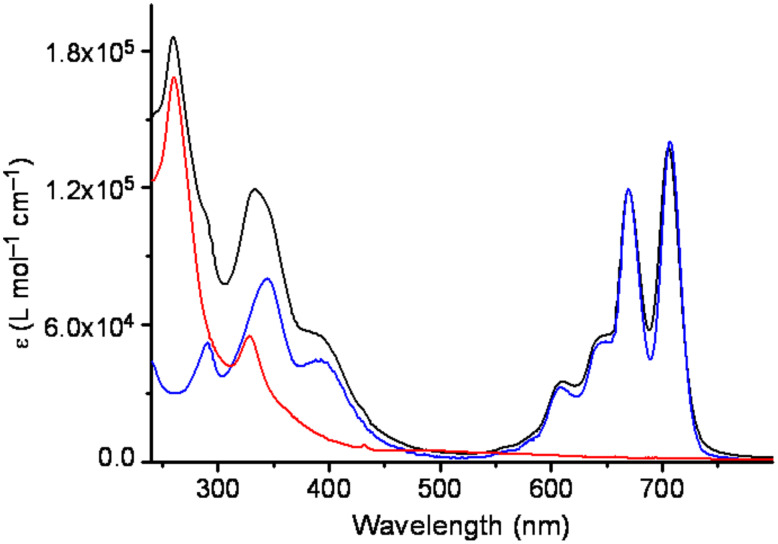
UV–vis absorption spectra of **2a** (black), **4a** (blue) and **1** (red) in CH_2_Cl_2_.

The structure of the phthalocyanines **4a–d** was confirmed by ^1^H and ^13^C NMR, and HR-MALDI mass spectrometry. ^1^H NMR spectra of low-symmetry phthalocyanines **4a–d** and **10a–d** in CDCl_3_ closely resemble those of **3** and feature a series of three unstructured multiplets from the protons of 1,2,4-substituted benzene rings. Inner-core protons in **4a–d** and **10a–d** are observed as a broad and concentration-sensitive high-field signal. It should be noted that **3** (as well as any phthalocyanine derivative prepared from a 4-substituted phthalonitrile) represents a mixture of four regioisomers [48,53], while lower-symmetry **4a–d** and **10a–d** are actual mixtures of as many as eight regioisomers each. This greatly complicates their NMR spectra (see Supporting Information). Furthermore, each regioisomer is a mixture of different diastereoisomers due to the presence of the asymmetric carbon atom in every peripheral substituent. However, although 2-decyltetradecyl substituents in **3** and **10a–d** are formally chiral, they should actually be treated as pseudo-achiral since the difference between the two of the substituents at the asymmetric carbon (the two linear alkyl groups of different length) is very small. As was shown previously, the complex diastereochemical composition does not influence the behavior of phthalocyanines bearing similar swallow-tail branched alkyl chains [46,54–57]. UV–vis absorption spectra of **4a–d** (Figure 2) are characteristic for metal-free phthalocyanine derivatives: they feature two intense long-wave absorption bands (termed Q-bands).

In the final synthetic step, reaction between **4a–d** and the acid **11** (prepared by acid-catalyzed hydrolysis of the commercially available methyl ester **1**, PCBM) [10,58] afforded the corresponding dyads **2a–d** (Scheme 2). Although in general esterification appears as a trivial synthetic transformation, esterification of **11** is greatly complicated by its poor solubility in virtually all organic solvents. After some experimenting, we found that the classical dicyclohexylcarbodiimide/*N*,*N*-dimethylaminopyridine (DCC/DMAP) protocol gives the best results, provided the acid **11** was first sonicated in *o*-dichlorobenzene for two hours prior to reaction, and then an alcohol **4a–d**, DCC and DMAP were added to the reaction mixture. After purification by column chromatography, the dyads **2a–d** were isolated in yields up to 45%. Again, the relatively modest yields are comparable with or superior to those previously reported for the esterification of poorly soluble acid **11** with various alcohols [22,50,59].

The structure of dyads **2a–d** was confirmed by NMR, MALDI MS and UV–vis absorption spectroscopy. ^1^H NMR spectra of the dyads **2a–d** essentially correspond to the superposition of the spectra of the phthalocyanine **4a–d** and PCBM (**1**), with the exception of the signal of CH_2_OC=O in dyads **2a–d** (ca. 4.10 ppm), which is shifted downfield vs. the signal of C*H*_2_OH in phthalocyanines **4a–d** (ca. 3.80 ppm). ^13^C NMR spectrum of the dyad **2c** also essentially represents a superposition of the spectra of phthalocyanine **4c** and PCBM (**1**). However, its detailed interpretation is greatly complicated because of a large number of signals, broadening of the signals due to the complex isomeric composition of phthalocyanine (see above), and overlaps of signals (see Supporting Information for the original NMR spectra). For this reason, only selected ^13^C chemical shifts are given in the Experimental Part. UV–vis absorption spectra of the dyads **2a–d** also appear as superposition of the spectra of **4a–d** and **1**: next to the intense Q-band (maxima at 670 and 707 nm) and B-band (between ca. 300 and ca. 450 nm) of phthalocyanine moiety, very strong high energy absorption of the cyclopropanated C_60_ derivative with the maximum at ca. 260 nm is observed (Figure 2).

**Figure 3:**
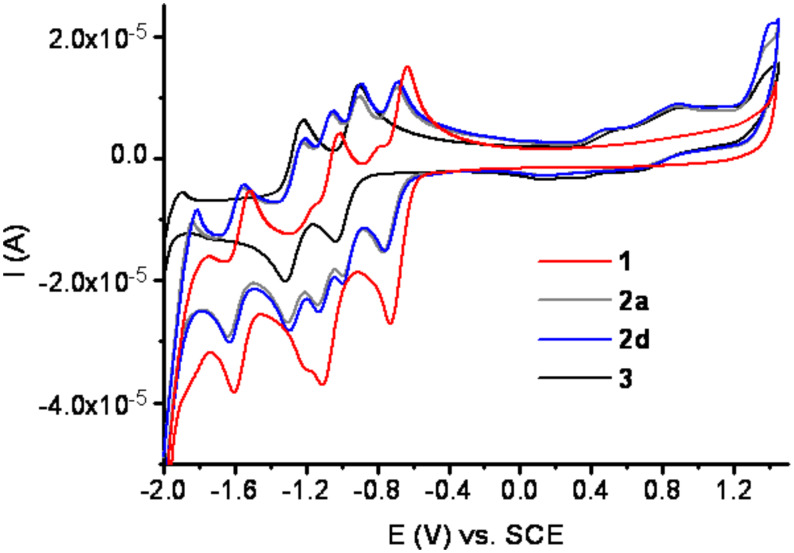
Cyclic voltammograms of **1** (red), **2a** (grey), **2d** (blue) and **3** (black) in CH_2_Cl_2_ (*c* = 10^−4^ M), scan rate 100 mV s^−1^.

Cyclic voltammetry measurements of dyads **2a** and **2d** in CH_2_Cl_2_ produced essentially identical results. Wave potentials do not change upon elongation of the spacer between the donor and the acceptor (Table 1). Furthermore, the voltammograms effectively correspond to the superposition of those for PCBM (**1**) and the phthalocyanine **3**, and do not display supplementary waves (Figure 3). The reduction part of the voltammograms of **2a**,**d** displays five quasi-reversible reduction waves, three due to the C_60_ moiety and two due to the phthalocyanine fragment. In the oxidation part of the voltammograms of **2a**,**d**, several poorly resolved waves identical to those of phthalocyanine **3** are observed at potentials up to +1.6 V vs. saturated calomel electrode (SCE). These observations, together with UV–vis spectra, suggest the absence of intramolecular charge transfer in the ground state for dyads **2a–d**.

**Table 1:** Experimental values of reduction potentials E^i^_red_ = (Ep^i^_c_ + Ep^i^_a_)/2 of **1**, **2a,d** and **3,** vs. SCE, in CH_2_Cl_2_ (*c* = 10^−4^ M), scan rate 100 mV s^−1^.

	E^1^_red_, V	E^2^_red_, V	E^3^_red_, V	E^4^_red_, V	E^5^_red_, V

**1**	−0.68		−1.06		−1.67
**2a**	−0.73	−0.94	−1.09	−1.26	−1.60
**2d**	−0.71	−0.94	−1.09	−1.25	−1.59
**3**		−0.96		−1.27	

Preliminary investigation of the dyads **2a–d** by polarized optical microscopy (POM) showed that the length of the spacer between the phthalocyanine and the fullerene moieties significantly affects the thermotropic properties of the material. Dyads **2a,b** are liquid at room temperature, while dyads **2c,d** display at room temperature fluidic birefringent textures confirming the occurrence of liquid crystalline (LC) mesophases. Upon heating, transitions to isotropic liquid were observed at 120 °C and 90 °C for **2c** and **2d**, respectively. For **2c** this transition was only detectable by POM and not by differential scanning calorimetry (DSC), while for **2d** DSC on heating revealed an endothermic peak at 90 °C [enthalpy 2.7 kJ/mol] in perfect agreement with POM observation. The small enthalpy value supports the transition from LC mesophase to isotropic liquid. However, assignment of the nature of LC mesophases for **1c,d** from the available data was not obvious, since the textures observed by POM were not characteristic. The major conclusion is nonetheless that the length of the spacer in **2a–d** between the C_60_ fragment and the mesogenic phthalocyanine moiety decisively affects the supramolecular structure of the material.

## Conclusion

We report the first example, to the best of our knowledge, of mesogenic phthalocyanine-C_60_ dyads. The key step in their synthesis is the preparation of the low-symmetry phthalocyanines, bearing three mesogenic swallow-tail substituents and an OH-terminated polymethylene linker. These key intermediates were synthesized by experimentally simple statistical condensation of two phthalonitriles, giving comparatively high yields (up to 24%) for this type of reaction. Upon the favorable combination of the length of the linker with the length of peripheral substituents on the phthalocyanine, bulky C_60_ moiety can be accommodated in the LC mesophase. Detailed analysis of this supramolecular organization, as well as deeper insight in the structure-property relationships of dyads **2**, will be the subject of a separate publication. Other objectives of our ongoing research include: studies of macroscopic alignment in films of dyads **2**, studies of charge carrier mobility in aligned films, and fabrication and evaluation of photovoltaic devices.

## Experimental

**General details.** All chemicals were purchased from Aldrich, Acros or TCI and used without further purification. Acid **11** was prepared according to the published procedure [10]. TLC was performed on precoated plates with silica gel 60 F_254_ (Merck), visualization with UV (λ = 254 nm). Column chromatography was performed on silica gel (0.040–0.063 mm, Macherey–Nagel). ^1^H NMR and ^13^C NMR spectra were recorded on Bruker Avance 300 or Varian VNMRS 400 spectrometers; chemical shifts are given in ppm relative to Me_4_Si (internal standard); coupling constants *J* are given in Hz. MALDI mass spectra were recorded on a Waters MALDI-QTOF Premier, using a 350 mW laser with dithranol (1,8-dihydroxy-10*H*-anthracen-9-one) as matrix for phthalocyanines and with DCB (*trans*-2-[3-(4-tert-butylphenyl)-2-methyl-2-propenylidene]malonitrile) as matrix for fullerene derivatives; EI and ESI mass spectra were recorded on a Waters AutoSpec 6. UV–Visible absorption spectra were recorded on a HP 8453 spectrophotometer in a quartz cell (optical path of 1 cm). For POM experiments, a NIKON Eclipse 80i microscope equipped with a digital camera DS Camera Head D5-5M was used; temperature was controlled by a Linkam Scientific Instruments GS350 hot stage. For DSC, a Perkin Elmer Diamond DSC calorimeter was used.

**Electrochemistry.** Cyclic voltammetry experiments were performed with a computer controlled Autolab potentiostat. Measurements were carried out at room temperature in a three-electrode single-compartment cell (10 mL), in CH_2_Cl_2_ solutions (concentration 10^−4^ M), containing Bu_4_NPF_6_ (0.1 M) as supporting electrolyte, at a scan rate of 100 mV s^−1^. A glassy carbon, polished by a slurry-suspension of alumina on micro-cloth and washed by Milli-Q water before each experiment, was used as a working electrode. A spiral platinum wire was employed as counter electrode and an Ag/AgCl/KCl(sat) used as reference electrode was connected to the cell solution via a salt bridge containing a saturated aqueous KCl solution. The Ag/AgCl electrode was checked against the ferrocene/ferrocinium (Fc/Fc^+^) couple (E°_Fc/Fc+_ = 0.425 V vs. Ag/AgCl) before and after each experiment. All potentials are reported versus saturated calomel electrode (SCE) (E°_Fc/Fc+_ = 0.405 V vs. SCE). Before each measurement, solutions were deaerated by 20 min nitrogen bubbling.

**4-(2-Decyltetradecyloxy)phthalonitrile (5).** To a solution of phenol **7** (1.80 g, 12.5 mmol) and bromide **8** (4.18 g, 10 mmol) in dry DMF (60 mL) was added dry K_2_CO_3_ (1.72 g, 12.5 mmol). The mixture was stirred at 90 °C under Ar for 7 h. After cooling to r.t., the mixture was poured into water (60 mL) and extracted with AcOEt (3 × 200 mL). The combined organic layers were washed with aqueous NaHCO_3_ (5%), dried with MgSO_4_ and concentrated in vacuum. Column chromatography (SiO_2_, CH_2_Cl_2_) afforded pure **5** as a light yellow solid (4.81 mg, 80%); R_f_ 0.7 (CH_2_Cl_2_). Analytical data identical to those previously reported [46].

**4-{10-[(2-Methoxyethoxy)methoxy]decyloxy}phthalonitrile (9c).** A solution of phthalonitrile **6c** (570 mg, 1.47 mmol) and *i*-Pr_2_EtN (0.77 mL, 4.5 mmol) in CH_2_Cl_2_ (30 mL) was treated dropwise with MEMCl (0.33 mL, 2.94 mmol) and the resulting mixture was stirred overnight at r.t. The mixture was then treated with aqueous saturated NaHCO_3_ (35 mL); the organic layer was separated, the aqueous layer was extracted with CH_2_Cl_2_ (2 × 40 mL), combined organic layers were dried over MgSO_4_ and evaporated. Column chromatography of the residue (CH_2_Cl_2_/AcOEt 4 : 1, R_f_ 0.69) afforded **9c** (479 mg, 84%) as a light yellow solid; mp 43 °C; R_f_ 0.64 (CH_2_Cl_2_/AcOEt 9 : 1); ^1^H NMR (300 MHz, CDCl_3_, 25 °C): δ = 7.68 (d, ^3^*J*_H,H_ = 8.8 Hz, 1H), 7.24 (d, ^4^*J*_H,H_ = 2.6 Hz, 1H), 7.14 (dd, ^3^*J*_H,H_ = 8.8 Hz, ^4^*J*_H,H_ = 2.6 Hz, 1H), 4.69 (s, 2H, OCH_2_O), 4.03 (t, ^3^*J*_H,H_ = 6.5 Hz, 2H, CH_2_O), 3.68 (t, ^3^*J*_H,H_ = 5.1 Hz, 2H, CH_2_O), 3.52–3.60 (m, 4H, OCH_2_CH_2_O), 3.38 (s, 3H, MeO), 1.81 (quint, ^3^*J*_H,H_ = 7.2 Hz, 2H), 1.26–1.64 (m, 14H); ^13^C NMR (100 MHz, CDCl_3_, 25 °C): δ = 162.2, 135.1, 119.5, 119.3, 117.4, 115.7, 115.3, 107.0, 95.5, 71.8, 69.3, 67.9, 66.7, 59.0, 29.7, 29.4, 29.4, 29.4, 29.2, 28.7, 26.2, 25.8; HR-MS (EI): *m/z* calcd. for C_22_H_32_N_2_O_4_ ([M]^+^): 388.2362, found 388.2374.

**2-{10-[(2-Methoxyethoxy)methoxy]decyloxy}-9(10),16(17),23(24)-tri(2-decyltetradecyloxy)-29*H*,31*H*-phthalocyanine (10c).** Li (125 mg, 17.7 mmol) was dissolved under Ar in *n*-pentanol (10 mL) and the mixture was heated to reflux until Li was completely dissolved. After cooling to r.t., a solution of phthalonitriles **5** (3.41 g, 7.1 mmol) and **9c** (305 mg, 0.79 mmol) in *n*-pentanol (15 mL) was added dropwise and the mixture was heated to reflux overnight. After cooling to r.t., AcOH was added dropwise until pH 5–6 was reached. The green solid was filtered and extensively washed with MeOH. Column chromatography of the residue afforded first **3** (eluted with CH_2_Cl_2_, 1.76 g, 50%) and then **10c** (eluted with CH_2_Cl_2_/AcOEt 1 : 1, 347 mg, 24%). Green solid; R_f_ 0.75 (CH_2_Cl_2_/AcOEt 1:1). ^1^H NMR (300 MHz, CDCl_3_, 25 °C): δ = 8.50–8.90 (m, 4H), 8.10–8.50 (m, 4H), 7.30–7.60 (m, 4H), 4.71 (s, 2H, OCH_2_O), 4.25–4.50 (m, 8H, ArOC*H*_2_), 3.65–3.80 (m, 4H, C*H*_2_OCH_2_OC*H*_2_), 3.58–3.63 (m, 2H, OCH_2_C*H*_2_O), 3.36 (s, 3H, MeO), 2.10–2.23 (m, 5H, ArOCH_2_C*H*_2_ + ArOCH_2_C*H*), 1.15–1.90 (m, 134H), 0.85 (t, ^3^*J*_H,H_ = 6.8 Hz, 18H), −2.84 (s, 2H, NH); ^13^C NMR (100 MHz, CDCl_3_, 25 °C): δ = 160.2 (br), 147.0 (br), 136.6 (br), 127.8 (br), 122.2 (br), 117.0 (br), 104.0 (br), 94.6 (OCH_2_O), 70.9 (br, CH_2_O), 70.7 (br, CH_2_O), 67.4 (br, CH_2_O), 66.9 (CH_2_O), 65.7 (CH_2_O), 58.0 (MeO), 37.5 (br), 30.5–31.0 (several CH_2_), 28.3–29.4 (several CH_2_), 26.3 (CH_2_), 25.2–25.5 (several CH_2_), 21.5–21.8 (several CH_2_), 13.1 (Me); HR-MS (MALDI): *m/z* calcd. for C_118_H_190_N_8_O_7_ ([M]^+^): 1831.4757, found 1831.4748.

**2-(10-Hydroxy-decyloxy)-9(10),16(17),23(24)-tri(2-decyltetradecyloxy)-29*H*,31*H*-phthalocyanine (4c).** A stirred solution of the phthalocyanine **10c** (275 mg, 0.15 mmol) and PPTS (188 mg, 0.75 mmol) in *t*-BuOH (20 mL) was heated to reflux overnight. After cooling to r.t., the reaction mixture was concentrated in vacuum. The resulting solid was suspended in MeOH (50 mL), then filtered, extensively washed with MeOH and dried to give **4c** (261 mg, 100%). Green solid; ^1^H NMR (300 MHz, CDCl_3_, 25 °C): δ = 8.90–9.20 (m, 4H), 8.45–8.80 (m, 4H), 7.50–7.75 (m, 4H), 4.35–4.55 (m, 8H, ArOC*H*_2_), 3.72 (t, ^3^*J*_H,H_ = 6.0 Hz, 2H, C*H*_2_OH), 2.03–2.20 (m, 5H, ArOCH_2_C*H*_2_ + ArOCH_2_C*H*), 1.10–1.95 (m, 134H), 0.80–0.93 (m, 18H), −3.07 (s, 2H, NH); ^13^C NMR (100 MHz, CDCl_3_, 25 °C): δ = 161.0 (br), 147.6 (br), 137.4 (br), 128.6 (br), 123.0 (br), 118.2 (br), 104.8 (br), 71.6 (br, CH_2_O), 68.4 (br, CH_2_O), 63.1 (CH_2_O), 38.5 (br), 32.9 (br), 31.6–32.2 (several CH_2_), 29.4–30.5 (several CH_2_), 27.3 (CH_2_), 26.3–26.6 (several CH_2_), 25.9, 22.6–22.9 (several CH_2_), 14.2 (Me); HR-MS (MALDI): *m/z* calcd. for C_114_H_182_N_8_O_5_ ([M]^+^): 1743.4233, found 1743.4240.

**Phthalocyanine-C_60_ dyad 2c.** A solution of the phthalocyanine **4c** (64 mg, 0.037 mmol) and the acid **11** (50 mg, 0.056 mmol) in *o*-dichlorobenzene (7 mL) was sonicated for 2 h. After cooling to 0 °C, DCC (31 mg, 0.15 mmol) and DMAP (18 mg, 0.15 mmol) were added, the mixture was allowed to reach r.t. and was stirred overnight. The resulting mixture was concentrated and the residue was purified by column chromatography (CH_2_Cl_2_/hexane 3 : 2) to give **2c** (46 mg, 47%). Green solid; R_f_ 0.67 (CH_2_Cl_2_/hexane 3 : 2); ^1^H NMR (300 MHz, CDCl_3_, 25 °C): δ = 8.25–8.80 (m, 4H), 7.90–8.20 (m, 4H), 7.72 (d, ^3^*J*_H,H_ = 7.4 Hz, 2H), 7.30–7.60 (m, 7H), 4.20–4.50 (m, 8H, ArOC*H*_2_), 4.05–4.15 (m, 2H, COOCH_2_), 2.67 (t, ^3^*J*_H,H_ = 7.5 Hz, 2H, CH_2_COO), 2.39 (t, ^3^*J*_H,H_ = 7.0 Hz, 2H, PhCC*H*_2_), 1.98–2.25 (m, 7H, ArOCH_2_C*H*_2_ + ArOCH_2_C*H* + C*H*_2_CH_2_COO), 1.10–1.90 (m, 134H), 0.80–0.95 (m, 18H), −3.36 (s, 2H, NH); ^13^C NMR (100 MHz, CDCl_3_, 25 °C), selected signals: δ = 173.1 (C=O), 160.3 (br, arom. C–O), 79.7 (fullerene C(sp^3^)), 71.8 (br, CH_2_O), 68.4 (br, CH_2_O), 64.7 (*CH*_2_OC=O), 51.7 (Ph*C*), 33.5 and 34.0 (*CH*_2_C=O and PhC*CH*_2_); HR-MS (MALDI): *m/z* calcd. for C_185_H_192_N_8_O_6_ ([M]^+^): 2621.4965 (M^+^), found 2621.5530.

## Supporting Information

Experimental procedures and analytical data for derivatives **2a,b,d**, **4a,b,d**, **6a–d**, **9a,b,d**, **10a,b,d**.

File 1 Experimental details
