# Supplementary material for: Synthesis of mesogenic phthalocyanine-C60 donor–acceptor dyads designed for molecular heterojunction photovoltaic devices
Source: Beilstein J Org Chem. 2009 Oct 7;5:49. doi: 10.3762/bjoc.5.49 (PMC2779692; doi:10.3762/bjoc.5.49)
Supplement: File 1 — Experimental details [file Beilstein_J_Org_Chem-05-49-s001.doc]

**Supporting Information**

**Synthesis of mesogenic phthalocyanine-C_60_ donor–acceptor dyads designed for molecular heterojunction photovoltaic devices**

Yves Henri Geerts^*1^, Olivier Debever^1^, Claire Amato^1^, Sergey Sergeyev^*1,2^

Address: ^1^Université Libre de Bruxelles (ULB), Laboratoire de Chimie des Polymères, CP 206/01, Boulevard du Triomphe, 1050 Bruxelles, Belgium ^2^University of Antwerp, Department of Chemistry, Groenenborgerlaan 171, 2020 Antwerp, Belgium

Email: Sergey Sergeyev - serguei.sergueev@ua.ac.be; Yves Henri Geerts - ygeerts@ulb.ac.be

**Description of Supporting Information**

1. Experimental details and spectra

2. Additional illustrations explaining the distribution of phthalocyanines resulting from the statistical condensations of phthalonitriles

3. Original ^1^H NMR spectra of **2c**, **4c**, and **1**

4.Original ^13^C NMR spectra of **2c**, **4c**, and **1**

5. Original MALDI-TOF spectra of **2c**

**4-(6-Hydroxyhexyloxy)phthalonitrile (6a).** Prepared similarly to **5** from 6-bromohexan-1-ol (290 mg, 1.6 mmol), **7** (460 mg, 2 mmol), and K_2_CO_3_ (360 mg, 2.6 mmol) in dry DMF (15 mL). Yield 336 mg (86%). Light yellow solid; mp 65 °C; R_f_ = 0.41 (CH_2_Cl_2_); ^1^H NMR (300 MHz, CDCl_3_, 25 °C): δ = 7.74 (d, ^3^*J*_H,H_ = 8.8 Hz, 1H), 7.30 (d, ^4^*J*_H,H_ = 2.6 Hz, 1H), 7.24 (dd, ^3^*J*_H,H_ = 8.8 Hz, ^4^*J*_H,H_ = 2.6 Hz, 1H), 4.09 (t, ^3^*J*_H,H_ = 6.4 Hz, 2H, *CH_2_*OAr), 3.67 (t, ^3^*J*_H,H_ = 6.6 Hz, 2H, *CH_2_*OH), 1.86 (quint, ^3^*J*_H,H_ = 6.7, 2H), 1.37–1.67 (m, 6H), 1.33 (br, 1H, OH); ^13^C NMR (75 MHz, CDCl_3_, 25 °C): δ = 162.1, 135.2, 119.4, 119.3, 117.4, 115.7, 115.2, 107.0, 69.1, 62.7, 32.7, 28.7, 25.6, 25.4; HR-MS (EI): *m/z* calcd. for C_14_H_16_N_2_O_2_ ([M]^+^): 244.1212, found: 244.1205.

**4-(8-Hydroxyoctyloxy)phthalonitrile (6b).** Prepared similarly to **6a** from 8-bromooctan-1-ol (334 mg, 1.6 mmol). Yield 375 mg (81%). Light yellow solid; mp 61 °C; R_f_ 0.40 (CH_2_Cl_2_); ^1^H NMR (300 MHz, CDCl_3_, 25 °C): δ = 7.68 (d, ^3^*J*_H,H_ = 8.7 Hz, 1H), 7.25 (d, ^4^*J*_H,H_ = 2.5 Hz, 1H), 7.18 (dd, ^3^*J*_H,H_ = 8.8 Hz, ^4^*J*_H,H_ = 2.6 Hz, 1H), 4.03 (t, ^3^*J*_H,H_ = 6.5 Hz, 2H, *CH_2_*OAr), 3.59 (t, ^3^*J*_H,H_ = 6.6 Hz, 2H, *CH_2_*OH), 1.79 (quint, ^3^*J*_H,H_ = 6.9 Hz, 2H), 1.32–1.61 (m, 10H), signal of OH was not detected due to overlap with other signals; ^13^C NMR (75 MHz, CDCl_3_, 25 °C): δ =  162.0, 135.0, 119.5, 119.2, 116.9, 115.6, 115.2, 106.4, 69.1, 62.4, 32.3, 29.0, 28.9, 28.4, 25.5, 25.4; HR-MS (EI): *m/z* calcd. for C_16_H_20_N_2_O_2_ ([M]^+^): 272.1525, found 272.1534.

**4-(10-Hydroxydecyloxy)phthalonitrile (6c).** Prepared similarly to **6a** from 10-bromodecan-1-ol (380 mg, 1.6 mmol). Yield 399 mg (83%). Light yellow solid; mp 59 °C; R_f_ 0.43 (CH_2_Cl_2_); ^1^H NMR (300 MHz, CDCl_3_, 25 °C): δ = 7.68 (d, ^3^*J*_H,H_ = 8.8 Hz), 7.24 (d, ^4^*J*_H,H_ = 2.6 Hz), 7.16 (dd, ^3^*J*_H,H_ = 8.8 Hz, ^4^*J*_H,H_ = 2.6 Hz, 1H), 4.03 (t, ^3^*J*_H,H_ = 6.4 Hz, 2H, *CH_2_*OAr), 3.63 (t, ^3^*J*_H,H_ 6.6 Hz, 2H, *CH_2_*OH), 1.81 (quint, ^3^*J*_H,H_ = 6.7 Hz, 2H), 1.23–1.63 (m, 14H), signal of OH was not detected due to overlap with other signals; ^13^C NMR (75 MHz, CDCl_3_, 25 °C): δ =  162.1, 135.2, 119.4, 119.3, 117.4, 115.7, 115.2, 107.0, 69.1, 62.7, 32.7, 29.7, 29.5, 29.3, 29.2, 28.7, 26.2, 25.8; HR-MS (EI): *m/z* calcd. for C_18_H_24_N_2_O_2_ ([M]^+^): 300.1838, found 300.1845.

**4-[12-(Hydroxy)dodecyloxy]phthalonitrile (6d).** Prepared similarly to **6a** from 12-bromododecan-1-ol (424 mg, 1.6 mmol). Yield 420 mg (80%). Light yellow solid; mp 64 °C; R_f_ 0.44 (CH_2_Cl_2_); ^1^H NMR (300 MHz, CDCl_3_, 25 °C): δ = 7.68 (d, ^3^*J*_H,H_ *=* 8.8 Hz, 1H), 7.24 (d, ^4^*J*_H,H_ = 2.6 Hz, 1H), 7.16 (dd, ^3^*J*_H,H_ = 8.8 Hz, ^4^*J*_H,H_ = 2.6 Hz, 1H), 4.03 (t, ^3^*J*_H,H_ = 6.4 Hz, 2H, *CH_2_*OAr), 3.63 (t, ^3^*J*_H,H_ = 6.6 Hz, 2H, *CH_2_*OH), 1.81 (quint, ^3^*J*_H,H_ = 6.7 Hz, 2H), 1.23–1.63 (m, 18H), signal of OH was not detected due to overlap with other signals; ^13^C NMR (75 MHz, CDCl_3_, 25 °C): δ =  162.2, 135.4, 119.4, 119.2, 117.5, 115.6, 115.2, 107.1, 69.2, 62.7, 32.4, 29.7, 29.5, 29.4, 29.2, 28.7, 26.4, 26.2, 25.8, 25.7; HR-MS (EI): *m/z* calcd. for C_20_H_28_N_2_O_2_ ([M]^+^) 328.2151, found 328.2139.

**4-{6-[(2-Methoxyethoxy)methoxy]hexyloxy}phthalonitrile (9a).** Prepared similarly to **9c** from phthalonitrile **6a** (100 mg, 0.41 mmol), MEMCl (0.1 mL; 0.83 mmol) and *i*-Pr_2_EtN (0.17 mL, 1.00 mmol) in CH_2_Cl_2_ (9 mL). Yield 124 mg (91%). Column chromatography of the residue afforded **9a** (124 mg, 91%) as a light yellow solid; mp 46 °C; R_f_ 0.69 (CH_2_Cl_2_/AcOEt 4 : 1). ^1^H NMR (300 MHz, CDCl_3_, 25 °C): δ = 7.68 (d, ^3^*J*_H,H_ = 8.8 Hz, 1H), 7.24 (d, ^4^*J*_H,H_ = 2.6 Hz, 1H), 7.14 (dd, ^3^*J*_H,H_ = 8.8 Hz, ^4^*J*_H,H_ = 2.6 Hz, 1H), 4.69 (s, 2H, OCH_2_O), 4.03 (t, ^3^*J*_H,H_ = 6.5 Hz, 2H, CH_2_O), 3.68 (t, ^3^*J*_H,H_ = 5.0 Hz, 2H, CH_2_O), 3.52–3.60 (m, 4H, OCH_2_CH_2_O), 3.38 (s, 3H, MeO), 1.81 (quint, ^3^*J*_H,H_ = 7.2 Hz, 2H), 1.27–1.63 (m, 6H); ^13^C NMR (75 MHz, CDCl_3_, 25 °C): δ = 162.2, 135.1, 119.5, 119.3, 117.4, 115.7, 115.3, 107.0, 95.5, 71.8, 69.3, 67.9, 66.7, 59.0, 29.7, 29.4 , 28.7, 26.2; HR-MS (EI): *m/z* calcd. for C_18_H_24_N_2_O_4_ ([M]^+^) 332.1736, found 332.1721.

**4-{8-[(2-Methoxyethoxy)methoxy]octyloxy}phthalonitrile (9b).** Prepared similarly to **9c** from phthalonitrile **6a** (400 mg, 1.47 mmol), MEMCl (0.33 mL, 2.94 mmol) and *i*-Pr_2_EtN (0.77 mL, 4.5 mmol) in CH_2_Cl_2_ (30 mL). Yield 471 mg (89%). Light yellow solid; mp 41 °C; R_f_ 0.67 (CH_2_Cl_2_/AcOEt 4 : 1). ^1^H NMR (300 MHz, CDCl_3_, 25 °C): δ = 7.68 (d, ^3^*J*_H,H_ = 8.8 Hz, 1H), 7.24 (d, ^4^*J*_H,H_ = 2.6 Hz, 1H), 7.14 (dd, ^3^*J*_H,H_ = 8.8 Hz, ^4^*J*_H,H_ = 2.6 Hz, 1H), 4.69 (s, 2H, OCH_2_O), 4.03 (t, ^3^*J*_H,H_ = 6.5 Hz, 2H, CH_2_O), 3.68 (t, ^3^*J*_H,H_ = 5.0 Hz, 2H, CH_2_O), 3.52–3.60 (m, 4H, OCH_2_CH_2_O), 3.38 (s, 3H, MeO), 1.81 (quint, ^3^*J*_H,H_ = 7.2 Hz, 2H), 1.27–1.63 (m, 10H); ^13^C NMR (75 MHz, CDCl_3_, 25 °C): δ = 162.0, 135.0, 119.4, 119.1, 117.0, 115.6, 115.1, 106.5, 95.1, 71.6, 69.0, 67.6, 66.4, 58.7, 29.3, 29.0, 28.9, 28.4, 25.8, 25.4; HR-MS (ESI): *m/z* calcd. for C_20_H_28_N_2_O_4_ ([M+Na]^+^): 383.1947, found 383.1935.

**4-{12-[(2-Methoxyethoxy)methoxy]dodecyloxy}phthalonitrile (9d).** Prepared similarly to **9c** from phthalonitrile **6d** (285 mg, 0.74 mmol), MEMCl (0.17 mL, 1.47 mmol) and *i*-Pr_2_EtN (0.39 mL, 2.25 mmol) in CH_2_Cl_2_ (15 mL). Yield 286 mg (93%). Light yellow solid; mp 39 °C; R_f_ 0.62 (CH_2_Cl_2_/AcOEt 9 : 1); ^1^H NMR (300 MHz, CDCl_3_, 25 °C): δ = 7.68 (d, ^3^*J*_H,H_ = 8.8 Hz, 1H), 7.24 (d, ^4^*J*_H,H_ = 2.6 Hz, 1H), 7.14 (dd, ^3^*J*_H,H_ = 8.8 Hz, ^4^*J*_H,H_ = 2.6 Hz, 1H), 4.69 (s, 2H, OCH_2_O), 4.03 (t, ^3^*J*_H,H_ = 6.5 Hz, 2H, CH_2_O), 3.68 (t, ^3^*J*_H,H_ =5.0 Hz, 2H, CH_2_O), 3.52–3.60 (m, 4H, OCH_2_CH_2_O), 3.38 (s, 3H, MeO), 1.81 (quint, ^3^*J*_H,H_ = 7.2 Hz, 2H), 1.27–1.63 (m, CH_2_, 18H); ^13^C NMR (75 MHz, CDCl_3_, 25 °C): δ = 162.2, 135.1, 119.5, 119.3, 117.4, 115.7, 115.3, 107.0, 95.5, 71.8, 69.3, 67.9, 66.7, 59.0, 29.7, 29.4, 29.4, 29.4, 29.3, 29.3, 29.2, 28.7, 26.2, 25.8; HR-MS (ESI): *m/z* calcd. for C_24_H_36_N_2_O_4_ ([M+Na]^+^) 439.2573, found 439.2570.

**2-{6-[(2-Methoxyethoxy)methoxy]hexyloxy}-9(10),16(17),23(24)-tri(2-decyltetradecyloxy)-29*H*,31*H*-phthalocyanine (10a).** Prepared similarly to **10c** from **5** (2.93 g, 6.1 mmol), **9a** (225 mg, 0.68 mmol) and Li (105 mg, 15 mmol) in *n*-pentanol (15 mL). Yield of **10a** 157 mg (13%). Green solid; R_f_ 0.75 (CH_2_Cl_2_/AcOEt 1 : 1); ^1^H NMR (300 MHz, CDCl_3_, 25 °C): δ = 8.50–8.90 (m, 4H), 8.10–8.50 (m, 4H), 7.30–7.55 (m, 4H), 4.82 (s, 2H, OCH_2_O), 4.28–4.57 (m, 8H, ArOC*H*_2_), 3.67–3.80 (m, 4H, C*H*_2_OCH_2_OC*H*_2_), 3.58–3.63 (m, 2H, OCH_2_C*H*_2_O), 3.42 (s, 3H, MeO), 2.05–2.20 (m, 5H, ArOCH_2_C*H*_2_ + ArOCH_2_C*H*), 1.15–1.90 (m, 126H), 0.87 (t, ^3^*J*_H,H_ =7.0 Hz, 18H), –2.76 (br, 2H, NH); HR-MS (MALDI): *m/z* calcd. for C_114_H_182_N_8_O_7_ ([M]^+^) 1775.4132, found 1775.4167.

**2-{8-[(2-Methoxyethoxy)methoxy]octyloxy}-9(10),16(17),23(24)-tri(2-decyltetradecyloxy)-29*H*,31*H*-phthalocyanine (10b).** Prepared similarly to **10c** from **5** (3.41 g, 7.1 mmol), **9b** (284 mg, 0.79 mmol), Li (125 mg, 17.7 mmol), and pentanol (25 mL). Yield of **10b** 299 mg (21%). Green solid; R_f_ 0.75 (CH_2_Cl_2_/AcOEt 1 : 1). ^1^H NMR (300 MHz, CDCl_3_, 25 °C): δ = 8.50–8.90 (m, 4H), 8.10–8.50 (m, 4H), 7.30–7.60 (m, 4H), 4.82 (s, 2H, OCH_2_O), 4.28–4.54 (m, 8H, ArOC*H*_2_), 3.65–3.80 (m, 4H, C*H*_2_OCH_2_OC*H*_2_), 3.58–3.63 (m, 2H, OCH_2_C*H*_2_O), 3.45 (s, 3H, MeO), 2.07–2.20 (m, 5H, ArOCH_2_C*H*_2_ + ArOCH_2_C*H*), 1.15–1.90 (m, 130H), 0.92 (t, ^3^*J*_H,H_ =7.0 Hz, 18H), –2.91 (s, 2H, NH). HR-MS (MALDI): *m/z* calcd. for C_116_H_186_N_8_O_7_ ([M]^+^) 1803.4445, found 1803.4220

**2-{12-[(2-Methoxyethoxy)methoxy]dodecyloxy}-9(10),16(17),23(24)-tri(2-decyltetradecyloxy)-29*H*,31*H*-phthalocyanine (10d).** Prepared similarly to **10c** from **5** (3.41 g, 7.1 mmol), **9d** (330 mg, 0.79 mmol), Li (125 mg, 17.7 mmol), and pentanol (25 mL). Yield of **10d** 264 mg (18%). Green solid; R_f_ 0.75 (CH_2_Cl_2_/AcOEt 1 : 1). ^1^H NMR (300 MHz, CDCl_3_, 25 °C): δ = 8.50–8.90 (m, 4H), 8.10–8.50 (m, 4H), 7.30–7.60 (m, 4H), 4.76 (s, 2H, OCH_2_O), 4.25–4.52 (m, 8H, ArOC*H*_2_), 3.65–3.80 (m, 4H, C*H*_2_OCH_2_OC*H*_2_), 3.58–3.63 (m, 2H, OCH_2_C*H*_2_O), 3.45 (s, 3H, MeO), 2.10–2.23 (m, 5H, ArOCH_2_C*H*_2_ + ArOCH_2_C*H*), 1.15–1.90 (m, 138H), 0.88 (t, ^3^*J*_H,H_ = 6.8 Hz, 18H), –2.26 (s, 2H, NH); HR-MS (MALDI): *m/z* calcd. for C_120_H_194_N_8_O_7_ ([M]^+^) 1859.5071, found 1859.5079.

**2-(6-Hydroxyhexyloxy)-9(10),16(17),23(24)-tri(2-decyltetradecyloxy)-29*H*,31*H*-phthalocyanine (4a).** Prepared similarly to **4c** from **10a** (266 mg, 0.15 mmol). Yield 253 mg (100%). Green solid; ^1^H NMR (300 MHz, CDCl_3_, 25 °C): δ = 8.90–9.20 (m, 4H), 8.45–8.80 (m, 4H), 7.50–7.75 (m, 4H), 4.35–4.55 (m, 8H, ArOC*H*_2_), 3.78 (t, ^3^*J*_H,H_ = 6.0 Hz, 2H, C*H*_2_OH), 2.05–2.20 (m, 5H, ArOCH_2_C*H*_2_ + ArOCH_2_C*H*), 1.10–1.95 (m, 126H), 0.75–0.95 (m, 18H), NH not observed; HR-MS (MALDI): *m/z* calcd. for C_110_H_174_N_8_O_5_ (([M]^+^): 1687.3607, found 1687.3147.

**2-(8-Hydroxyoctyloxy)-9(10),16(17),23(24)-tri(2-decyltetradecyloxy)-29*H*,31*H*-phthalocyanine (4b).** Prepared similarly to **4a** from **10b** (270 mg, 0.15 mmol). Yield 257 mg (100%). Green solid; ^1^H-NMR (300 MHz, CDCl_3_, 25 °C): δ = 8.90–9.20 (m, 4H), 8.45–8.80 (m, 4H), 7.50–7.75 (m, 4H), 4.35–4.55 (m, 8H, ArOC*H*_2_), 3.80 (t, ^3^*J*_H,H_ = 6.0 Hz, 2H, C*H*_2_OH), 2.03–2.20 (m, 5H, ArOCH_2_C*H*_2_ + ArOCH_2_C*H*), 1.10–1.95 (m, 130H), 0.79–0.93 (m, 18H), –3.10 (s, 2H, NH); HR-MS (MALDI): *m/z* calcd. for C_112_H_178_N_8_O_5_ ([M]^+^): 1715.3920, found 1715.4406.

**2-(12-hydroxy-dodecyloxy)-9(10),16(17),23(24)-tri(2-decyltetradecyloxy)-29*H*,31*H*-phthalocyanine (4d).** Prepared similarly to **4a** from **10d** (280 mg, 0.15 mmol). Yield 266mg (100%); Green solid; ^1^H-NMR (300 MHz, CDCl_3_, 25 °C): δ = 8.90–9.20 (m, 4H), 8.45–8.80 (m, 4H), 7.50–7.75 (m, 4H), 4.35–4.55 (m, 8H, ArOC*H*_2_), 3.69 (t, ^3^*J*_H,H_ = 6.0 Hz, 2H, C*H*_2_OH), 2.03–2.20 (m, 5H, ArOCH_2_C*H*_2_ + ArOCH_2_C*H*), 1.10–1.95 (m, 138H), 0.80–0.93 (m, 18H), –2.97 (s, 2H, NH); HR-MS (MALDI): *m/z* calcd. for C_116_H_186_N_8_O_5_ ([M]^+^): 1771.4546, found 1771.4551.

**Phthalocyanine-C_60_ dyad 2a.** Prepared similarly to dyad **2c** starting from **4c** (63 mg, 0.037 mmol). Yield 43 mg (45%). Viscous green liquid; R_f_ 0.64 (CH_2_Cl_2_/hexane 3 : 2); ^1^H-NMR (300 MHz, CDCl_3_, 25 °C): δ = 8.25–8.80 (m, 4H), 7.90–8.20 (m, 4H), 7.79 (d, ^3^*J*_H,H_ = 7.4 Hz, 2H), 7.35–7.55 (m, 7H), 4.20–4.50 (m, 8H, ArOC*H*_2_), 4.05–4.15 (m, 2H, COOCH_2_), 2.68 (t, ^3^*J*_H,H_ = 7.5 Hz, 2H, CH_2_COO), 2.42 (t, ^3^*J*_H,H_ = 7.0 Hz, 2H, PhCC*H*_2_), 1.95–2.25 (m, 7H, ArOCH_2_C*H*_2_ + ArOCH_2_C*H* + C*H*_2_CH_2_COO), 1.15–1.95 (m, 126H), 0.80–0.95 (m, 18H), –3.20 (s, 2H, NH); HR-MS (MALDI): *m/z* calcd. for C_181_H_184_N_8_O_6_ (([M]^+^): 2565.4339, found 2565.4316.

**Phthalocyanine-C_60_ dyad 2b.** Prepared similarly to dyad **2a** starting from **4b** (64 mg, 0.037 mmol). Yield 40 mg (42%). Viscous green liquid; R_f_ 0.60 (CH_2_Cl_2_/hexane 3 : 2); ^1^H-NMR (300 MHz, CDCl_3_, 25 °C): δ = 8.25–8.80 (m, 4H), 7.90–8.20 (m, 4H), 7.70 (d, ^3^*J*_H,H_ = 7.4 Hz, 2H), 7.30–7.60 (m, 7H), 4.20–4.50 (m, 8H, ArOC*H*_2_), 4.05–4.15 (m, 2H, COOCH_2_), 2.64 (t, ^3^*J*_H,H_ = 7.5 Hz, 2H, CH_2_COO), 2.43 (t, ^3^*J*_H,H_ = 7.0 Hz, 2H, PhCC*H*_2_), 1.95–2.25 (m, 7H, ArOCH_2_C*H*_2_ + ArOCH_2_C*H* + C*H*_2_CH_2_COO), 1.15–1.90 (m, 130H), 0.80–0.95 (m, 18H), –3.20 (s, 2H, NH); HR-MS (MALDI): *m/z* calcd. for C_183_H_188_N_8_O_6_ ([M]^+^): 2593.4652, found: 2593.5417.

**Phthalocyanine-C_60_ dyad 2d**. Prepared similarly to dyad **2a** starting from **4d** (66 mg, 0.037 mmol). Yield 39 mg (40%). Green solid; R_f_ 0.65 (CH_2_Cl_2_/hexane 3:2); ^1^H NMR (300 MHz, CDCl_3_, 25 °C): δ = 8.25–8.80 (m, 4H), 7.90–8.20 (m, 4H), 7.78 (d, ^3^*J*_H,H_ = 7.4 Hz, 2H), 7.30–7.60 (m, 7H), 4.20–4.50 (m, 8H, ArOC*H*_2_), 4.05–4.15 (m, 2H, COOCH_2_), 2.72 (t, ^3^*J*_H,H_ = 7.5 Hz, 2H, CH_2_COO), 2.40 (t, ^3^*J*_H,H_ = 7.0 Hz, 2H, PhCC*H*_2_), 1.98–2.25 (m, 7H, ArOCH_2_C*H*_2_ + ArOCH_2_C*H* + C*H*_2_CH_2_COO), 1.10–1.90 (m, 138H), 0.80–0.95 (m, 18H), –2.95 (s, 2H, NH); HR-MS (MALDI): *m/z* calcd. for C_187_H_196_N_8_O_6_ ([M]^+^): 2649.5278, found 2649.5444.

**Figure SI1:** Statistical condensation of the two dinitriles A and B leads to a mixture of phthalocyanines A_4_, A_3_B, A_2_B_2_, AB_3_ and B_4_.


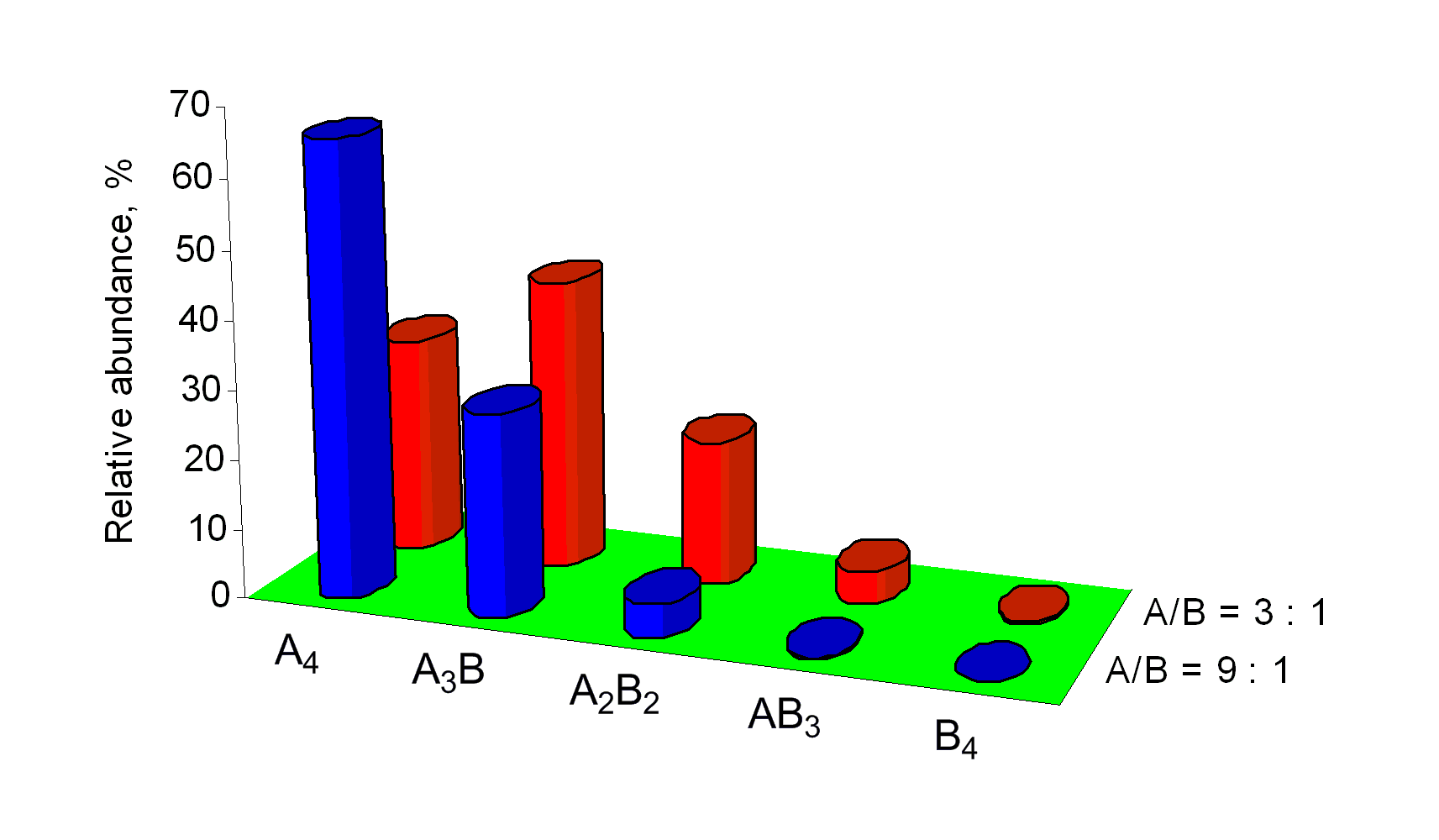


**Figure SI2:** The distribution of phthalocyanines A_4_, A_3_B, A_2_B_2_, AB_3_ and B_4_ resulting from statistical condensation of the two dinitriles A and B can be described by the coefficients of a binom (3A + B)^4^ = 81A^4^ + 108A^3^B + 54A^2^B^2^ + 12AB^3^ + B^4^ (for the molar ratio A/B = 3 : 1) or (9A + B)^4^ = 6561A^4^ + 2916A^3^B + 486A^2^B^2^ + 36AB^3^ + B^4^ (for the molar ratio A/B = 9 : 1).

**Figure SI3:** Condensation of 3-substituted phthalonitrile A produces a statistical mixture of four regioisomeric phthalocyanines of different symmetry (*C*_4_*_h_*, *D*_2_*_h_*, *C*_2_*_v_*, *C_s_*). Statistical condensation of the two 3-substituted dinitriles A and B produces A_3_B phthalocyanine as a mixture of eight regioisomers.


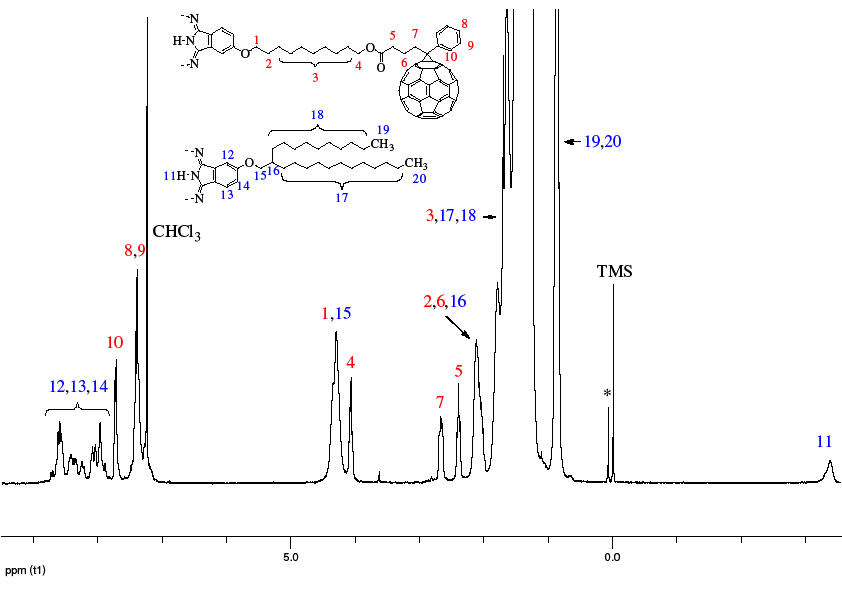


**Figure SI4:** ^1^H NMR spectrum of dyad **2c** (300 MHz, 25 °C, 7.6∙10^–3^ M).


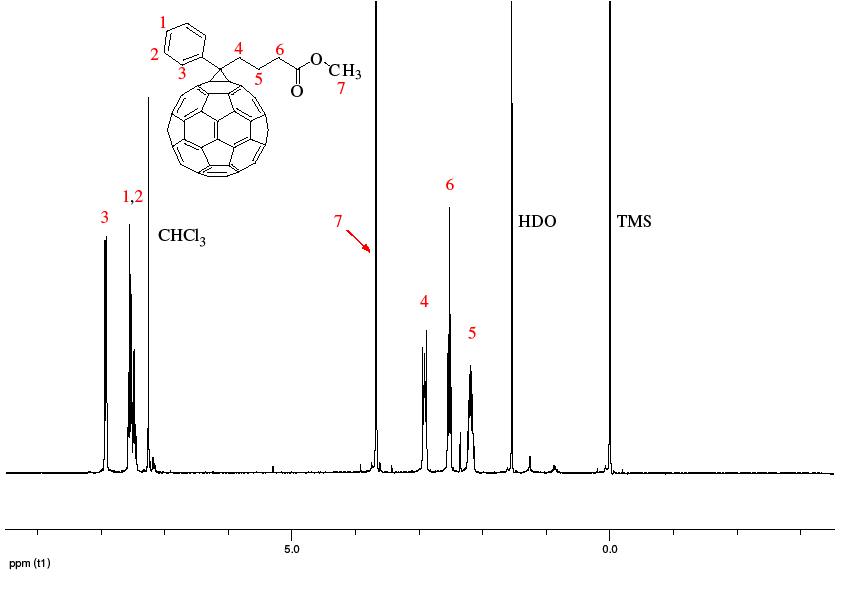


**Figure SI5:** ^1^H NMR spectrum of PCBM **1** (300 MHz, 25 °C, 2.2∙10^–2^ M).


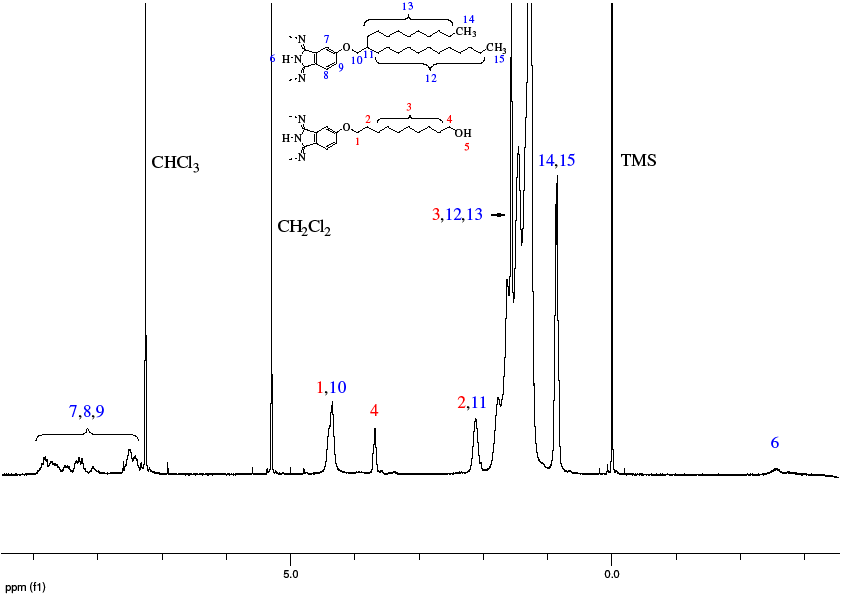


**Figure SI6:**^1^H NMR spectrum of phthalocyanine **4c** (300 MHz, 25°C, 1.15∙10^–3^ M).

**
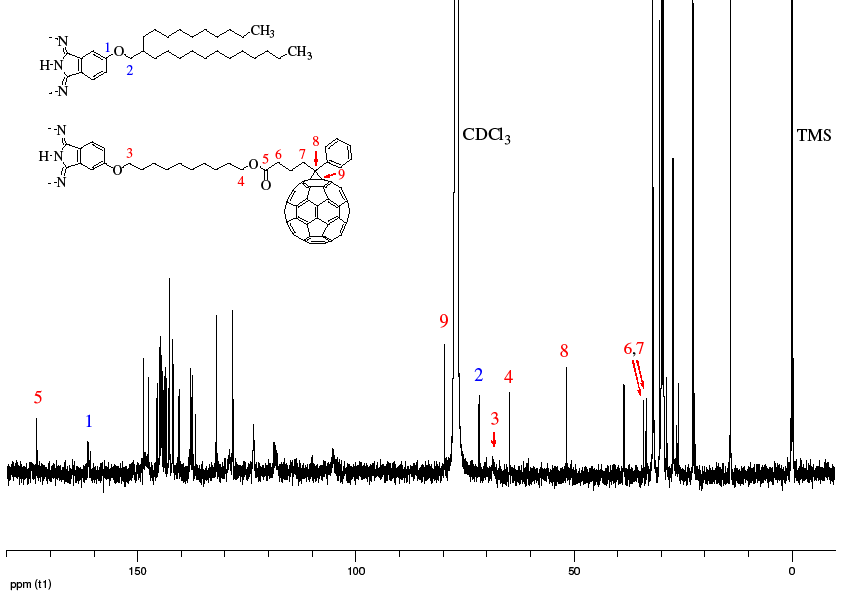
**

**Figure SI7:** ^13^C NMR spectrum of dyad **2c** (100 MHz, 25 °C, 3.05∙10^–2^ M).


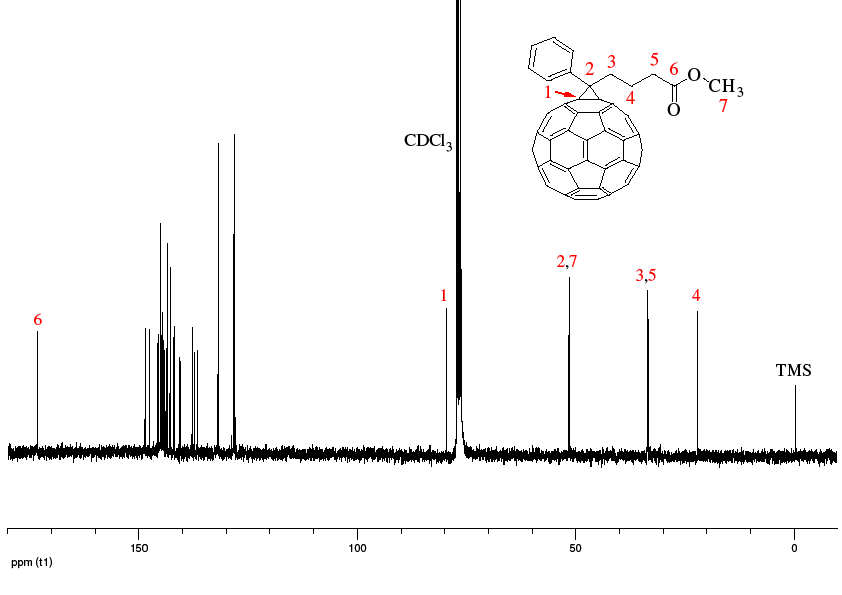


**Figure SI8:** ^13^C NMR spectrum of PCBM **1** (75 MHz, 25 °C, 2.2∙10^–2^ M).


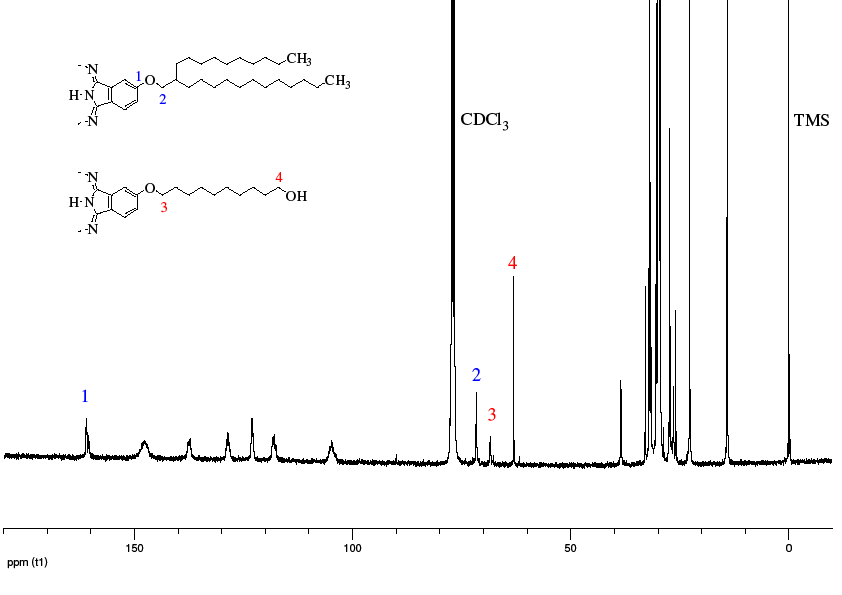


**Figure SI9:** ^13^C NMR spectrum of phthalocyanine **4c** (100 MHz, 25 °C, 4.6∙10^–2^ M).

**
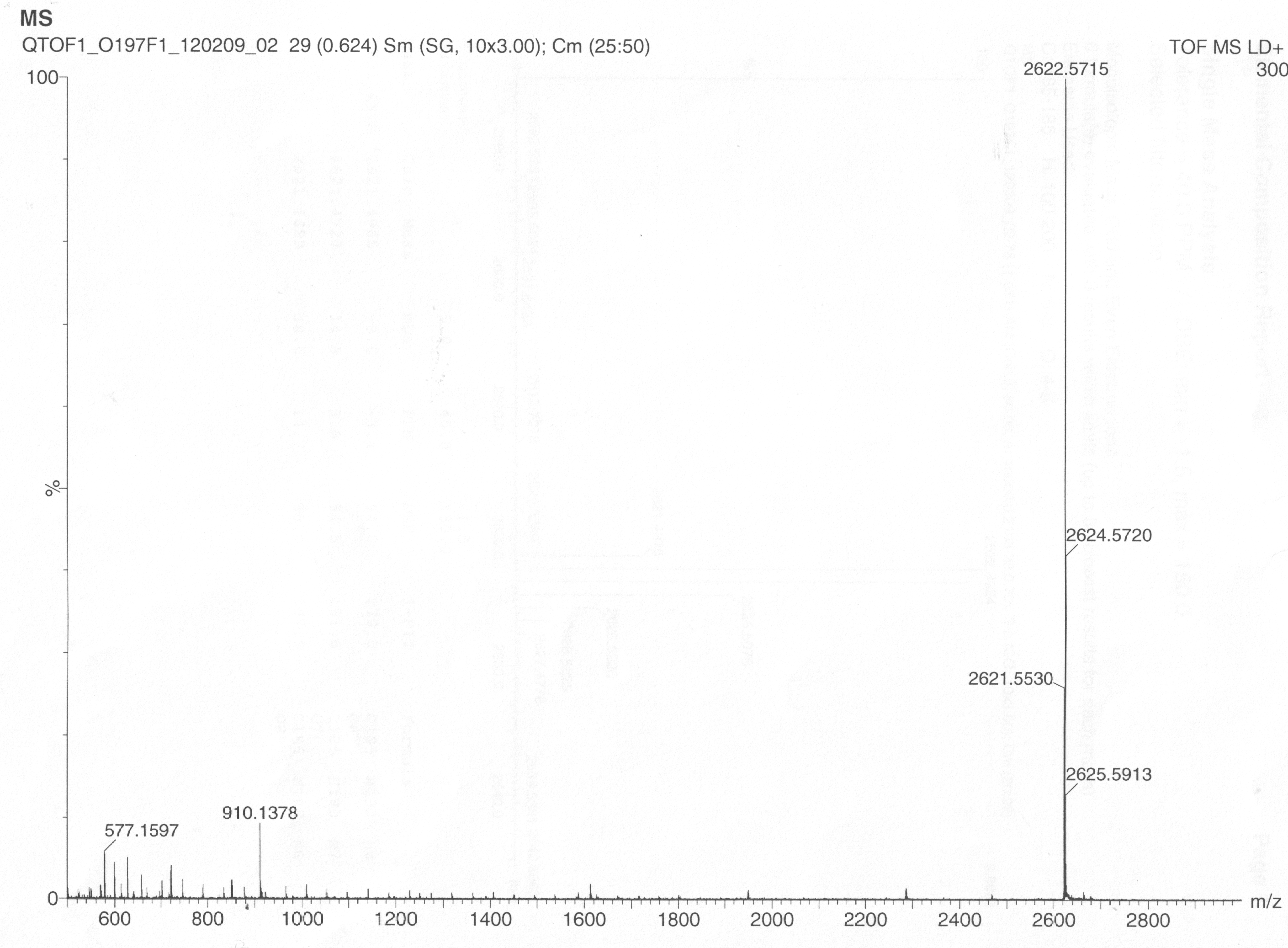
**

**Figure SI10:** MALDI-TOF mass spectrum of dyad **2c**.
